# Real-Time Microscopic Monitoring of Flow, Voltage and Current in the Proton Exchange Membrane Water Electrolyzer

**DOI:** 10.3390/s18030867

**Published:** 2018-03-15

**Authors:** Chi-Yuan Lee, Shih-Chun Li, Chia-Hung Chen, Yen-Ting Huang, Yu-Syuan Wang

**Affiliations:** 1 Department of Mechanical Engineering, Yuan Ze Fuel Cell Center, Yuan Ze University, Taoyuan 320, Taiwan; s1065007@mail.yzu.edu.tw (S.-C.L.); s1045020@mail.yzu.edu.tw (Y.-T.H.); s1040935@mail.yzu.edu.tw (Y.-S.W.); 2 HOMYTECH Global CO., LTD, Taoyuan 334, Taiwan; sv3@homytech.com

**Keywords:** PEM water electrolyzer, MEMS, flexible integrated microsensor, real-time microscopic monitoring

## Abstract

Looking for alternative energy sources has been an inevitable trend since the oil crisis, and close attentioned has been paid to hydrogen energy. The proton exchange membrane (PEM) water electrolyzer is characterized by high energy efficiency, high yield, simple system and low operating temperature. The electrolyzer generates hydrogen from water free of any carbon sources (provided the electrons come from renewable sources such as solar and wind), so it is very clean and completely satisfies the environmental requirement. However, in long-term operation of the PEM water electrolyzer, the membrane material durability, catalyst corrosion and nonuniformity of local flow, voltage and current in the electrolyzer can influence the overall performance. It is difficult to measure the internal physical parameters of the PEM water electrolyzer, and the physical parameters are interrelated. Therefore, this study uses micro-electro-mechanical systems (MEMS) technology to develop a flexible integrated microsensor; internal multiple physical information is extracted to determine the optimal working parameters for the PEM water electrolyzer. The real operational data of local flow, voltage and current in the PEM water electrolyzer are measured simultaneously by the flexible integrated microsensor, so as to enhance the performance of the PEM water electrolyzer and to prolong the service life.

## 1. Introduction

Hydrogen is very sparse in the earth’s atmosphere, and it mostly exists as compounds, such as water (H_2_O), natural gas (CH_4_) and so on. It is stored in a near limitless quantity in sea water and is the most abundant and environmentally clean alternative fuel with potentially lower costs than nonrenewable carbon-based fossil fuels [1]. Therefore, hydrogen will be produced. Furthermore, it is likely to replace fossil fuels in the future owing to the political, financial and environmental factors associated with the latter [2]. At present, hydrogen is mainly derived from fossil material recombination, coal gasification, industrial byproducts and water electrolysis. The latest hydrogen production methods include photocatalyst water decomposition, biomass pyrolysis, microbial fermentation and high temperature water pyrolysis. The water electrolyzing hydrogen production technique is limited to the power source and electrolytic efficiency; in order to reduce the energy consumption and to increase the production rate, the proton exchange membrane water electrolyzer is emerged accordingly [3]. In comparison to traditional water electrolysis, the proton exchange membrane water electrolyzer has high energy efficiency, high yield, high purity, simple system and low operating temperature; hydrogen can be separated from oxygen to avoid them mixing with each other which is dangerous [4]. However, after long-term operation of the proton exchange membrane water electrolyzer, the membrane material durability, catalyst corrosion and the nonuniform local flow, voltage and current in the electrolyzer can influence the overall performance. If the voltage is too high, the catalyst is destroyed by electrochemical corrosion, and if the voltage is too low the reaction is incomplete. The flow and electric current are the key factors determining the local performance [5,6,7,8,9,10]. The current measuring method is to connect the wire to the anode and the cathode of the collector plate, and measure the voltage distribution of the proton exchange membrane water electrolyzer under different operations. However, this method is an external measurement and cannot measure the internal voltage. Therefore, this study uses micro-electro-mechanical system (MEMS) technology to develop a flexible integrated microsensor. Internal multiple physical information is extracted to determine the optimal working parameters for the PEM water electrolyzer. 

## 2. Design of a Flexible Integrated Microsensor

### 2.1. Micro Flow Sensor

**Figure 1:**
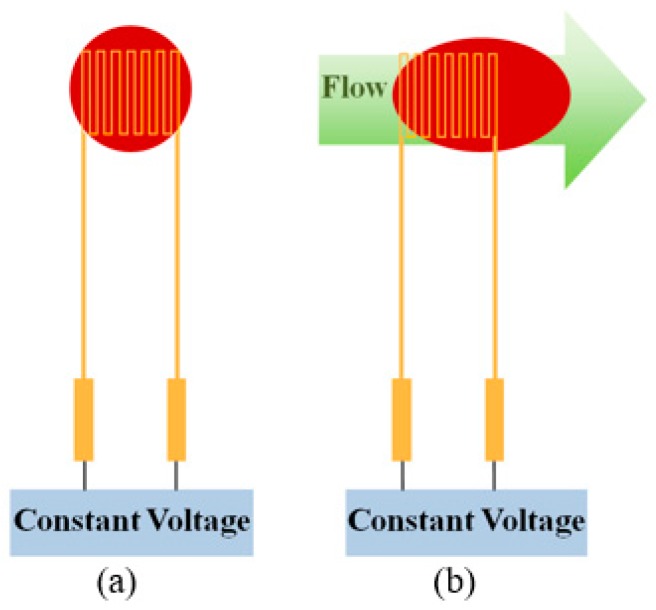
Sensing principle of a micro flow sensor: (**a**) no fluid through; (**b**) fluid through.

The main measurement structure of a hot-wire micro flow sensor is a resistance heater. A stable temperature field is generated by feeding a constant voltage, as shown in Figure 1a. When the fluid passes by the micro flow sensor, the temperature field is fixed with the heat of the heater, and the resistivity of the heater decreases as the fluid flow increases. The heat supplied to the hot wire is controlled, so that the temperature difference between the hot wire and flow is fixed; the heating power increases with fluid flow. The flow is converted into an electric signal and exported by constant temperature circuit design, varying with the forced convection of fluid, as shown in Figure 1b. 

In the heat transfer process, the heat transfer reaction on the hot wire surface is very complex, so there is not yet a specific computing equation for establishing the relationship between the average flow velocity and the complex reaction on the hot wire surface. Therefore, in this study, the hot wire current value was extracted directly to establish the relationship between the current value and liquid flow, so as to effectively reduce the errors in the equipment accuracy and computational process [11].

### 2.2. Micro Voltage Sensor

**Figure 2:**
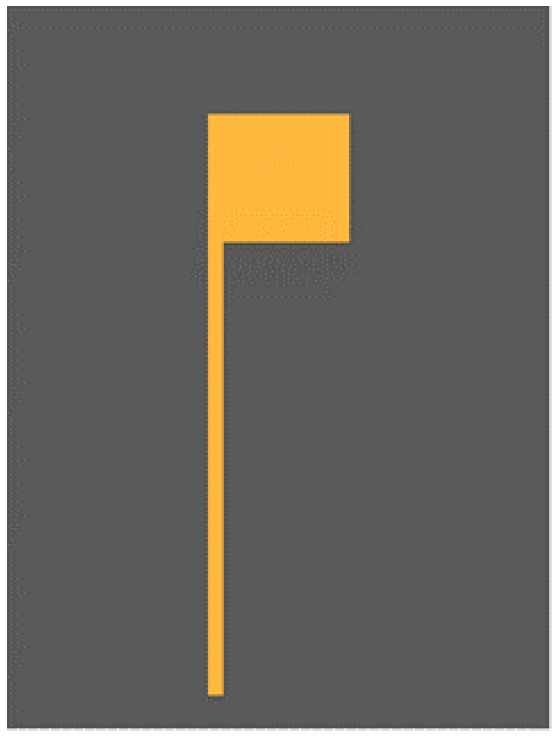
Schematic diagram of the micro voltage sensor.

The micro voltage sensor structure used in this study is an extension conductor; a conductor structure is formed on the substrate surface by thin film deposition and the lithography process. A 300 μm × 300 μm sensing area is exposed at the foremost end of the conductor as the probe of the voltmeter; the rest is insulated by insulating material, so as to make sure that the voltage extracted by the flaky probe is from the foremost end of the conductor when the conductor is embedded in the proton exchange membrane water electrolyzer. Figure 2 is the schematic diagram of the micro voltage sensor.

### 2.3. Micro Current Sensor

**Figure 3:**
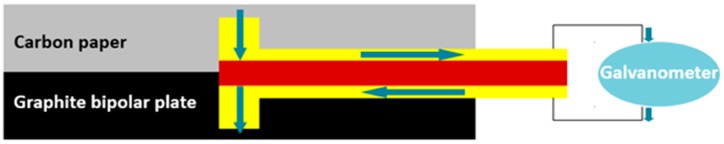
Schematic diagram of the principle of the micro current sensor.

The micro current sensor used in this study is a miniaturized galvanometer probe of an extension conductor. Two probes face towards the carbon paper and graphite plate respectively, and a 300 μm × 300 μm sensing area is exposed at the foremost end; the rest of the conductor is obstructed by an insulating layer; the local current is conducted to the instrument outside the proton exchange membrane water electrolyzer for measurement, as shown in Figure 3.

### 2.4. Integrated Design of a Flexible Integrated Microsensor

**Figure 4:**
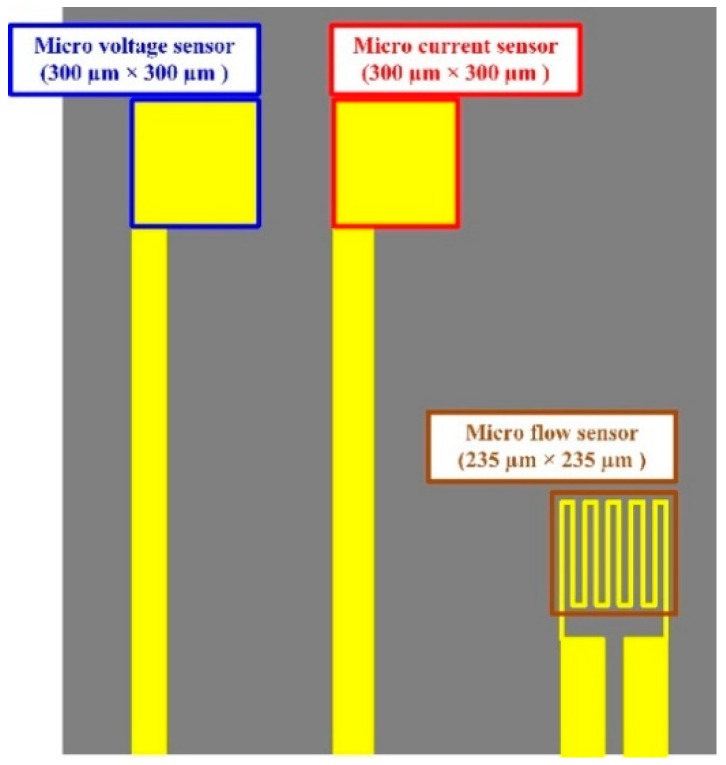
Design drawing of a flexible integrated microsensor.

Figure 4 shows the size design of the flexible integrated microsensor in this study, integrated with the flow, voltage and current sensing structure. The flow sensing area is 235 μm × 235 μm, the minimum line width is 10 μm; the voltage sensing area is 300 μm × 300 μm; the current sensing area is 300 μm × 300 μm. This integrated design method not only minimizes the coverage area of the microsensor in the proton exchange membrane water electrolyzer, but also minimizes the effect on the performance of the proton exchange membrane water electrolyzer, and the flow, voltage and current can be measured simultaneously.

## 3. Integration of the Proton Exchange Membrane Water Electrolyzer and a Flexible Integrated Microsensor

Three flexible integrated microsensors are embedded in the anode runner plate of the proton exchange membrane water electrolyzer in this study.

### 3.1. Correction of Micro Flow Sensor 

The flow is corrected by using a Germany KNF STEPDOS 03RC diaphragm liquid metering pump to provide steady flow. The diaphragm liquid metering pump uses vertical link motion to drive the diaphragm, so the diaphragm causes pressure difference in the pump head, leading to the material convey in or out. The flow range of STEPDOS 03RC is 0.03~30 mL/min; the flow accuracy is ±2%. 

**Figure 5:**
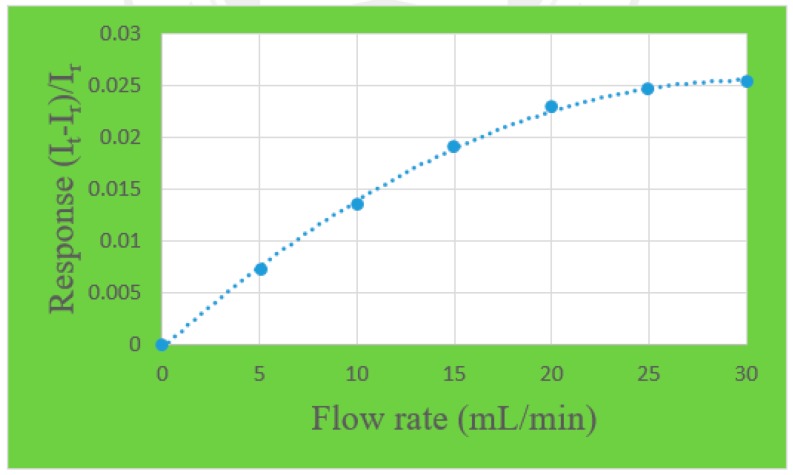
Correction curve of a micro flow sensor.

A one-runner graphite plate must be made before the micro flow sensor is corrected. The width and depth of the graphite plate are the same as the proton exchange membrane water electrolyzer. Afterwards, the micro flow sensor is embedded in the runner. Finally, the power supply, micro flow sensor and NI PXI 2575 data capture equipment are connected. The power supply supplies a constant voltage to generate a stable temperature field; the pure water measures the current variation value for flow calibration. The flow correction range is 0~30 mL/min, the unit of interval is 5, and there are seven signals extracted. The correction curve is shown in Figure 5.

### 3.2. Activation of the Proton Exchange Membrane Water Electrolyzer 

**Figure 6:**
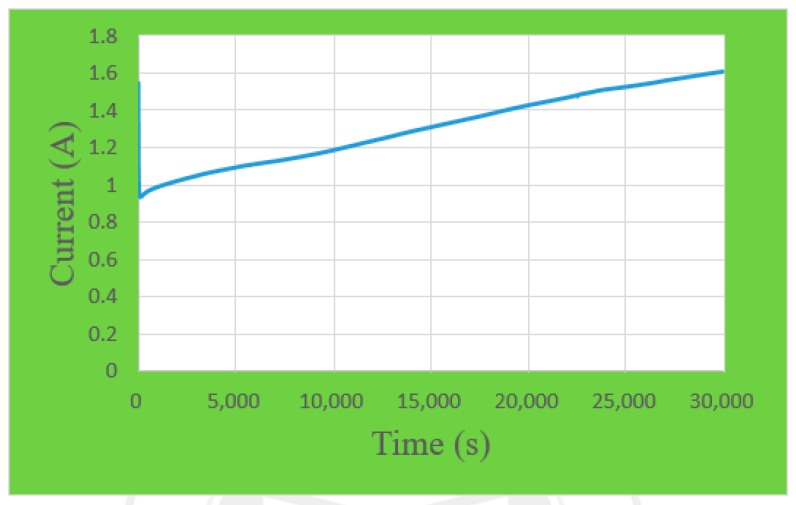
Activation curve diagram of the proton exchange membrane water electrolyzer.

The purpose of activation is to attain the required performance of the proton exchange membrane water electrolyzer, so its characteristics must be known before the actual measurement of the proton exchange membrane water electrolyzer. Figure 6 shows the activation process completed after 8 h operation under a set point voltage of 2.5 V.

## 4. Real-Time Microscopic Monitoring in the Proton Exchange Membrane Water Electrolyzer

The flexible integrated microsensor and high precision capture equipment NI PXI 2575 are used for local real-time microscopic monitoring in the proton exchange membrane water electrolyzer.

### 4.1. Flow Test 

**Figure 7:**
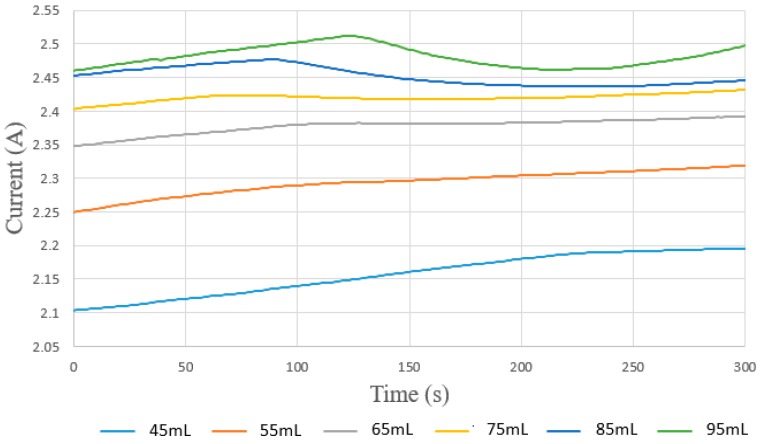
Performance curves of different flow rates.

The magnitude of flow determines the rate of electrochemical reaction in the proton exchange membrane water electrolyzer: the higher the flow, the faster the electrochemical reaction. However, the catalyst and membrane electrode assembly(MEA) may be damaged if the flow is too high, so the flow range is set as 45~95 mL/min in this study. The flow is tested six times at intervals of 10ml, the voltage is set as 2.5 V, the temperature is 90 °C. Figure 7 shows the test result.

### 4.2. Local Flow Distribution of the Proton Exchange Membrane Water Electrolyzer 

**Figure 8:**
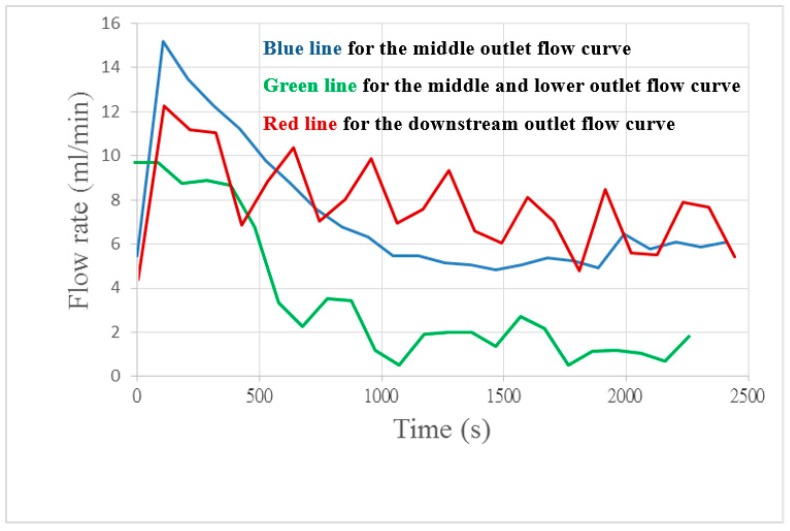
Flow curve diagram of different runners.

Figure 8 shows internal channels embedded in the downstream, midstream and upper midstream outlets. The initial flow of the runner is a little higher than the average flow which may be due to the fluid pressure drop; the fluid is stabilized with time.

### 4.3. Local Voltage Distribution of the Proton Exchange Membrane Water Electrolyzer 

**Figure 9:**
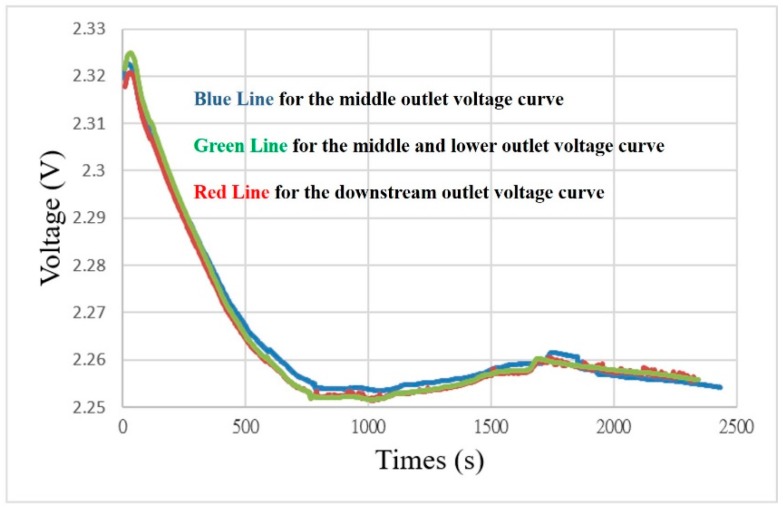
Voltage curve diagram of different runners.

An external constant voltage must be supplied in the operation of the proton exchange membrane water electrolyzer; the operating voltage is set as 2.5 V in this study. Figure 9 shows that the measured voltage is lower than 2.5 V which may be due to the external conductor and the resistance of the proton exchange membrane water electrolyzer.

### 4.4. Local Current Density Distribution of the Proton Exchange Membrane Water Electrolyzer 

**Figure 10:**
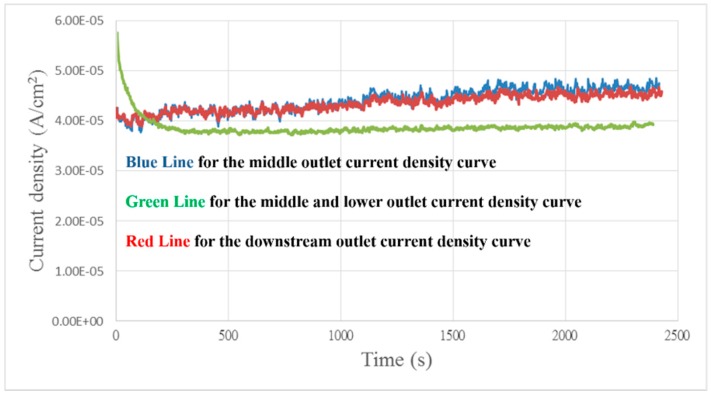
Current density curve diagram of different runners.

Figure 10 shows that the local current density at the downstream and midstream outlets is higher than at the upstream outlet which may be due to adequate flow through the downstream and midstream outlets. The MEA can perform a full reaction.

## 5. Conclusions

This study uses MEMS technology to integrate a micro flow, voltage and current sensor with a 50 μm thick polyimide (PI) film substrate, and uses electrochemical corrosion-resistant PI (Fujifilm Durimide^®^ PI 7320) as a protection layer. This flexible integrated microsensor is characterized by corrosion resistance, small area, high sensitivity, real-time measurement and arbitrary placement. 

The flexible integrated microsensor can be embedded in the anode runner plate without influencing the sealing condition of the proton exchange membrane water electrolyzer. The internal local flow, voltage and current information of the proton exchange membrane water electrolyzer is extracted successfully by an NI PXI 2575 data acquisition unit in the operation process of the proton exchange membrane water electrolyzer. The internal condition of proton exchange membrane water electrolysis is mastered and the parameters are adjusted, so as to enhance the performance and prolong the service life.
